# Supplementary material for: Self-testing knowledge and beliefs on HIV self-testing use in central Uganda
Source: PLOS Glob Public Health. 2024 Jun 12;4(6):e0002869. doi: 10.1371/journal.pgph.0002869 (PMC11168646; doi:10.1371/journal.pgph.0002869)
Supplement: S1 Text — Instrument measuring HST knowledge, Beliefs, and use. (DOCX) [file pgph.0002869.s001.docx]

Dear participant,

You are invited to participate in this study which focuses on Self-testing knowledge and beliefs on HIV self-testing use in central Uganda. Your participation will be kept anonymous and confidential. Work through the items below quickly. You should often go with your first instinctive response that comes to your mind when you read the item. Findings will be reported in a journal article.

**Nsereko Gerald Mukisa** has described to me what is going to be done, the risks, the benefits involved and my rights regarding this study. I understand that my decision to participate in this study does not alter my usual medical care. In the use of this information, my identity will be concealed. I am aware that I can withdraw at any time. I understand that by signing this form, I do not waive any of my legal rights but merely indicate that I have been informed about the research study in which I am voluntarily agreeing to participate.

Signature/thumb print ……………………………….. Date ………....................................

Name of person obtaining informed consent **Nsereko Gerald Mukisa**

Signature of interviewer……………………………………. Date... ………………….

**SECTION A: KNOWLEDGE OF HIVST**

*On a scale of 1 to 5, Please fill the boxes on the right by rating each statement according to your knowledge of HIVST using the scale in the table below.*

| Strongly Disagree (SD) | Disagree (D) | Neutral (N) | Agree (A) | Strongly Agree (SA) |
| --- | --- | --- | --- | --- |
| 1 | 2 | 3 | 4 | 5 |

| **SN.** | **Items** | **SD** | **D** | **N** | **A** | **SA** |
| --- | --- | --- | --- | --- | --- | --- |
|  | I know what HIVST is |  |  |  |  |  |
|  | I have heard about HIVST |  |  |  |  |  |
|  | I have read about HIVST |  |  |  |  |  |
|  | I have ever seen an HIVST kit |  |  |  |  |  |
|  | I have ever used an HIVST kit |  |  |  |  |  |
|  | It is legal to use an HIVST kit in Uganda |  |  |  |  |  |
|  | HIVST kits are available in private pharmacies |  |  |  |  |  |
|  | HIVST kits are available in government clinics and hospitals |  |  |  |  |  |
|  | HIVST kits are available on the internet |  |  |  |  |  |
|  | HIVST can be done using blood |  |  |  |  |  |
|  | HIVST can be done using saliva from the mouth |  |  |  |  |  |
|  | A person can perform HIVST on himself/herself |  |  |  |  |  |
|  | It takes 20 to 40 minutes to get results from HIVST process |  |  |  |  |  |
|  | The HIVST result can be negative if the HIV infection is less than 3 months old |  |  |  |  |  |
|  | A person needs to retest after 3 months if the test is negative |  |  |  |  |  |
|  | There is need for an HIV counselor before taking the HIV self-test |  |  |  |  |  |

**SECTION B: INDIVIDUAL BELIEFS**

*On a scale of 1 to 5, Please fill the boxes on the right by rating rate each statement according to your beliefs regarding HIVST using the scale in the table below.*

| Strongly Disagree (SD) | Disagree (D) | Neutral (N) | Agree (A) | Strongly Agree (SA) |
| --- | --- | --- | --- | --- |
| 1 | 2 | 3 | 4 | 5 |

| **SN.** | **Items** | **Scale** | | | | |
| --- | --- | --- | --- | --- | --- | --- |
| **Perceived susceptibility** | | ***SD*** | ***D*** | ***N*** | ***A*** | ***SA*** |
|  | I am afraid that I might contract HIV |  |  |  |  |  |
|  | I believe that I might get HIV even if I am having sex with only one partner |  |  |  |  |  |
|  | I believe that I might be infected with HIV if my sex partner is having unsafe sex with others |  |  |  |  |  |
| **Perceived Benefits** | | | | | | |
|  | I believe that HIVST will provide me with the option to know my HIV status and get emotional relief |  |  |  |  |  |
|  | I feel that HIVST will help me plan to avoid infection in the future |  |  |  |  |  |
|  | HIVST provides me with the option to get early treatment before getting seriously sick |  |  |  |  |  |
|  | I believe that I can plan my future with full confidence by knowing my HIV status |  |  |  |  |  |
|  | I believe that HIVST would help me not to transmit HIV to others if in case I had HIV |  |  |  |  |  |
|  | I believe that HIVST helps me identify my sexual partner based on his/her HIV status |  |  |  |  |  |
| **Perceived Barriers** | | | | | | |
|  | I am afraid of hearing HIV-positive results by undergoing HIVST |  |  |  |  |  |
|  | I am afraid of the stigma attached to HIV positive result |  |  |  |  |  |
|  | I am afraid of separation from my friends and family due to my HIV-positive result |  |  |  |  |  |
|  | I am embarrassed to ask for an HIVST kit |  |  |  |  |  |
|  | I am afraid that I may lose my partner if my HIV self-test result turned out to be positive |  |  |  |  |  |
|  | I don’t want anyone to know that I’m sexually active/ at risk |  |  |  |  |  |
|  | I am afraid that people may talk about me if I go to a health facility/pharmacy to pick up an HIV self-test kit |  |  |  |  |  |

**SECTION C: HIVST USE**

*On a scale of 1 to 5, Please fill the boxes on the right by rating each statement according to how you find the use of HIVST using the scale in the table below.*

| Strongly Disagree (SD) | Disagree (D) | Neutral (N) | Agree (A) | Strongly Agree (SA) |
| --- | --- | --- | --- | --- |
| 1 | 2 | 3 | 4 | 5 |

| **SN.** | **Items** | **SD** | **D** | **N** | **A** | **SA** |
| --- | --- | --- | --- | --- | --- | --- |
|  | I can use HIVST after receiving the testing kit |  |  |  |  |  |
|  | I can confirm the results from the HIV self-test |  |  |  |  |  |
|  | I feel comfortable using the HIV self-test |  |  |  |  |  |
|  | I find the instructions on the HIVST kit easily understood |  |  |  |  |  |
|  | I would recommend HIVST to others |  |  |  |  |  |
|  | I think my family and friends would use HIVST |  |  |  |  |  |
|  | I’m comfortable asking my main partner to use HIVST |  |  |  |  |  |
|  | I’m comfortable asking a casual partner to use HIVST |  |  |  |  |  |
|  | I can discuss HIVST with my sexual partner upon receiving the test kit. |  |  |  |  |  |

**SECTION D: DEMOGRAPHIC CHARACTERISTICS**

*Fill the most appropriate choice from the items below*

| **SN.** | **Items and scoring** |
| --- | --- |
| **DC1.** | Gender: a) Male b) Female |
| **DC2.** | Age: 18-21 22-25 26-29 30-33 34-37 38-41 |
| **DC3.** | Relationship status: a) Not Dating b) Casually Dating c) Steady Relationship    d) Co-Habiting e) Married f) Separated g) Divorced |
| **DC4.** | Religion: a) Protestant b) Moslem c) Catholic d) Seventh-Day Adventist  e) Born Again f) Other |
| **DC5.** | Level of education: a) Primary b) Highschool c) Certificate d) Diploma  e) Other |
| **DC6.** | In the past 6 months have you been sexually active? a) Yes b) No |

Thank you so much for taking the time to contribute to the success of this study, God bless you
